# Recognising and managing childhood onset uveitis: a guide for primary care

**DOI:** 10.3399/bjgp23X735225

**Published:** 2023-09-29

**Authors:** Eleanor Kelly, En-min Choi, Harry Petrushkin, Salomey Kellett, Ameenat Lola Solebo

**Affiliations:** Moorfields Eye Hospital, London.; Newton Place Surgery, Faversham, Kent.; Moorfields Eye Hospital, London; Rheumatology Department, Great Ormond Street Hospital, London.; Population, Policy and Practice Department, UCL Great Ormond Street Institute of Child Health, London; National Institute for Health and Care Research (NIHR) Great Ormond Street Hospital Biomedical Research Centre, London.; NIHR clinician scientist and consultant ophthalmologist, Rheumatology Department, Great Ormond Street Hospital, London; Population, Policy and Practice Department, UCL Great Ormond Street Institute of Child Health, London; NIHR Great Ormond Street Hospital Biomedical Research Centre, London.

## INTRODUCTION

Uveitis describes a collection of rare disorders characterised by intraocular inflammation. Childhood onset disease is rare, with a minimum estimated incidence rate of 1 per 100 000 children,^1^ although this is likely to be a significant underestimation of true disease burden, with other population studies reporting a prevalence of 1 per 1000 children.^1^^,^^2^ Presentation most commonly occurs at age 2–5 years, with another smaller peak age of onset in mid- adolescence.^3^^,^^4^ The condition develops in isolation or in association with an underlying systemic inflammatory disorder, such as juvenile idiopathic arthritis (JIA).^5^ Uveitis can affect multiple parts of the eye but typically involves the anterior ocular chamber (anterior uveitis, previously termed ‘iritis’). The structural eye complications caused by uveitis include cataracts, calcified corneal opacities (band keratopathy), maculopathy, and glaucoma.^4^ Managing the disease in childhood usually requires the use of immunosuppressants to avoid these complications, and to spare the child from the adverse effects of the long- term use of topical and systemic steroids.^6^ A key predictor of poorer outcomes is delayed detection of uveitis in children.^4^

## WHY IS PROMPT DIAGNOSIS OF CHILDHOOD UVEITIS IMPORTANT?

Permanent visual loss in childhood uveitis occurs because of irreversible structural ocular damage caused by uncontrolled intraocular inflammation.^4^ When structural complications are treatable or reversible (for example, cataract), permanent visual loss can still occur because of the interruption to visual simulation during the sensitive period of neurodevelopment. Delays in managing eye disorders that occur during the first 8 years of life can result in lifelong poor vision due to abnormal maturation of cerebral visual pathways. This maldevelopment of vision is termed amblyopia (colloquially, ‘lazy eye’).^7^

## HOW DOES CHILDHOOD UVEITIS PRESENT?

While adult-onset anterior uveitis commonly presents with a sudden-onset, painful, photophobic red eye, childhood-onset disease may be more insidious.^4^ Children may be largely asymptomatic, or may struggle to articulate symptoms. For this reason, children known to be at risk of uveitis, such as those with JIA, undergo regular surveillance examinations for uveitis, with examinations every 3–4 months.^3^^,^^4^ When symptomatic, childhood uveitis typically presents with symptoms such as red eye, photophobia, floaters, and/or changes to vision.

## WHEN SHOULD A GP SUSPECT UVEITIS IN A CHILD WITH RED EYES?

**Figure 1.**
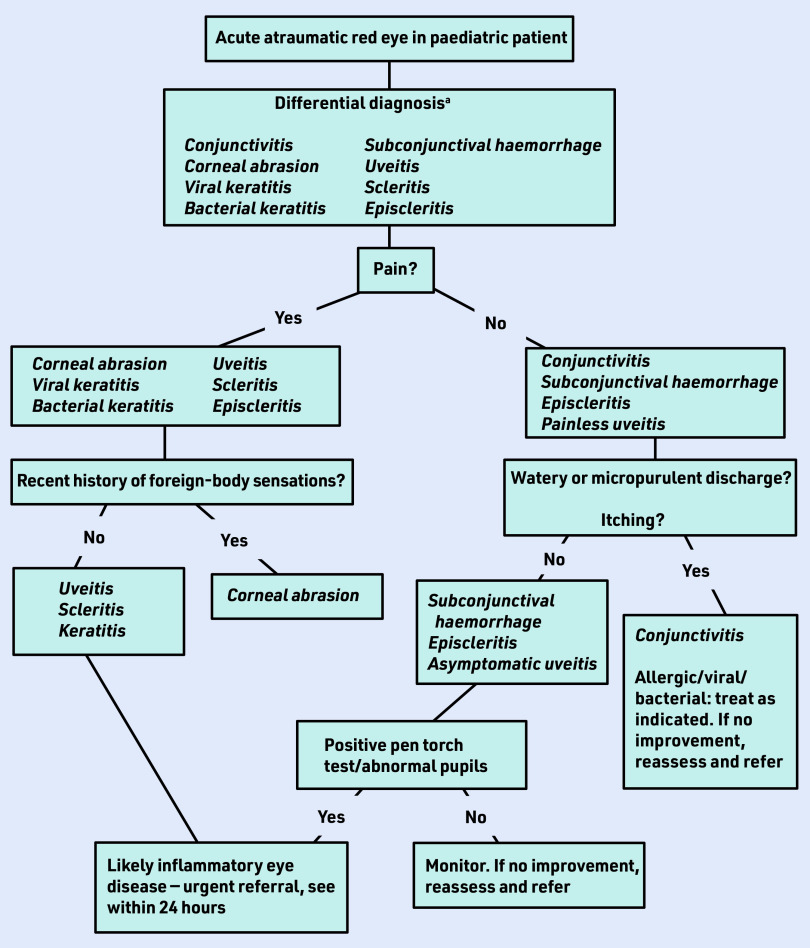
*Flow chart of diagnostic indicators for red eye in childhood.^a^ Subconjunctival haemorrhage — sharp defined edges, normal sclera surrounding it; episcleritis — unilateral superficial, moveable with a swab, non- painful; scleritis — characterised by severe pain and diffuse or focal redness.*

It may be a challenge to distinguish between uveitis and other causes of paediatric red eye.^8^ Figure 1 is a guide to when to suspect uveitis, or other serious ocular inflammatory conditions, in a paediatric patient.

**Figure 2.**
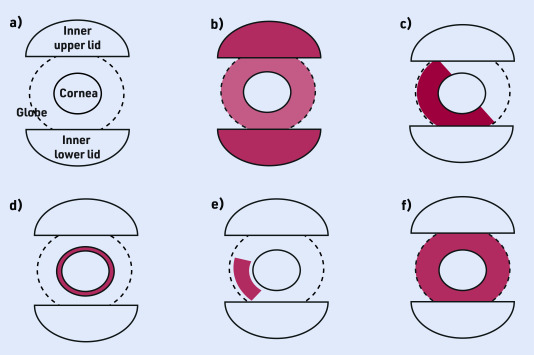
*Patterns of redness across different causes of red eye. Redness representing injection, haemorrhage, or other vascular change. a) Lids, globe, and cornea represented for healthy eye; b) redness primarily affecting tarsal (lid) conjunctiva, as seen in conjunctivitis; c) dark red sectoral change affecting globe or ‘bulb’ of eye, as seen in subconjunctival haemorrhage; d) injection primarily around corneal edge, also known as limbal area, as seen in corneal pathology (including keratitis) and in uveitis; e) sectoral injection, as seen in scleritis or episcleritis; and f) bulbar injection sparing the tarsal area, as seen in a diffuse scleritis or uveitis.*

Red flags in the child’s history and examination that should raise a suspicion of an inflammatory cause include:

- diagnoses of other autoimmune or autoinflammatory disorders (for example, JIA, psoriasis, and inflammatory bowel disorders) or a close family history of early-onset inflammatory disorders;
- new ocular structural changes (corneal scarring or irregularities of pupil colour or shape);
- ocular redness that spares the tarsal conjunctiva (Figure 2);
- photophobia (elicitation of pain on looking at bright light); and
- symptoms such as reduced vision and pain, which require an urgent referral for specialist review.

The pen torch test is a useful tool in differentiating between serious and non- serious unilateral red eyes. It is performed by shining a pen torch from a distance of 15 cm, into one eye, for 2 seconds.^9^ A positive result is when the patient reports any pain or discomfort, including mild responses, particularly in the contralateral (untested) eye. This test has a reported 80% positive predictive value.^9^ While assessing the eyes with a pen torch, the pupil characteristics can also be assessed. The parents of younger children should also be asked about avoidance of light or behaviours indicating discomfort with light.

## WHAT ROLE DOES PRIMARY CARE HAVE IN MANAGING CHILDHOOD UVEITIS?

Treatment is not expected to be initiated in primary care. At the initial visit to an ophthalmology department, active inflammation is graded. The child is then started on a tapering dose of topical steroids (oral steroids may be needed for severe disease) and undergoes systemic investigations as directed by the child’s history. A referral will also be made to general paediatrics or paediatric rheumatology. This is required not only to uncover underlying disorders, but is also necessary ahead of the start of systemic immunosuppressive agents.^6^ The most used first-line agents are methotrexate and mycophenolate, with second-line agents, such as the anti-TNF biologic immunomodulator adalimumab, added later if required. The use of these agents requires regular blood monitoring.^10^ This is typically full blood count (FBC), serum creatinine, and liver blood test (LFT) every 3 to 4 months. Children are seen frequently in specialist eye clinics, resulting in time away from school, and family disruption.^6^^,^^10^ Drug monitoring and re-prescription is often undertaken in primary or secondary care if considered appropriate, in order to support a more positive patient experience. The disease, and management of this chronic, relapsing remitting disease, carries a high burden on the child and family’s quality of life, with subsequent negative impact on mental health.^10^

In summary, although uveitis is uncommon in childhood, it is important to consider it as a potential diagnosis for a child presenting with a painful red eye or eyes, to ensure that appropriate urgent referral is made for swift management to prevent permanent sight loss. Primary care has a key role to play in the coordination of medical, psychological, and social care for the child with this chronic, complex disease, and the adult they become.
